# A Retrospective Analysis of Demographics, Clinical Features, and Treatment Patterns in Sickle Cell Disease Patients at a Tertiary Healthcare Centre of North East India

**DOI:** 10.7759/cureus.74489

**Published:** 2024-11-26

**Authors:** Anupam Dutta, Taniya S Dutta, Amlin Shukla, Papori Gogoi

**Affiliations:** 1 General Medicine, Assam Medical College and Hospital, Dibrugarh, IND; 2 Pediatrics, Assam Medical College and Hospital, Dibrugarh, IND; 3 Pediatrics, Indian Council of Medical Research, New Delhi, IND; 4 Medicine, Assam Medical College and Hospital, Dibrugarh, IND

**Keywords:** clinical features, hospital epidemiology, hydroxyurea therapy, north east region of india, sickle cell anaemia, sickle cell disease complications, sickle cell disease: scd

## Abstract

Background

Sickle cell disease (SCD) is a hereditary disorder marked by abnormal hemoglobin (HbS), leading to chronic hemolytic anemia, vaso-occlusive crises (VOCs), and multi-organ complications. In India, the prevalence of SCD is highest among tribal populations in states like Madhya Pradesh, Maharashtra, Odisha, and Assam, with the disease burden exacerbated by limited healthcare access, especially in rural regions. This study provides a comprehensive analysis of the demographic profile, clinical features, and treatment patterns of SCD patients at a tertiary healthcare center in Upper Assam, where the prevalence of SCD is high among the tea tribe communities.

Methods

This retrospective observational study included 250 patients diagnosed with various SCD subtypes who presented with SCD-related complications at Assam Medical College and Hospital between January 2020 and December 2023. Data were obtained from medical records in the departments of medicine and pediatrics, covering demographic variables (age, gender, ethnicity), clinical characteristics (complications, hemoglobin levels, genotype, history of hospitalizations), and treatment details (frequency and type of blood transfusions, use of hydroxyurea and chelating agents). Descriptive statistics summarized demographic and clinical features, while chi-square tests and t-tests were used for bivariate analysis. Logistic regression identified factors associated with high transfusion requirements.

Results

The study population had a mean age of 17.2 years, with 54.4% male predominance. Most patients (87%) had sickle cell anemia (HbSS), while the remainder had other genotypes including HbSA, HbSE, and sickle cell thalassemia. The most common presenting symptoms were fever (61.2%) and bone/joint pain (48.4%), indicative of VOCs and frequent infections. Pallor (30%) and abdominal pain (25.6%) were also prominent. Half of the patients (125) received hydroxyurea, though its uptake was limited by availability and cost. A high transfusion burden was noted, with 72.4% of patients requiring between five to 12 transfusions annually. However, only 22.4% received chelation therapy to manage iron overload, reflecting the cost constraints in accessing these agents. Laboratory findings indicated a mean hemoglobin level of 7.19 g/dL and elevated serum ferritin levels due to repeated transfusions. The frequency of blood transfusions was higher compared to Western studies, emphasizing the need for more accessible disease-modifying therapies in resource-limited settings.

Conclusions

The findings of this study illustrate the significant clinical and transfusion burden experienced by SCD patients in Upper Assam. This population relies heavily on blood transfusions due to limited access to hydroxyurea and other advanced therapies. The study underscores a critical need for improved access to hydroxyurea, expanded availability of chelation therapy, and greater healthcare support for managing SCD-related complications in resource-limited settings. As India’s National Sickle Cell Anemia Elimination Mission is implemented, regional studies such as this are essential for tailoring public health interventions to meet the specific needs of high-prevalence areas, especially among underserved tribal communities.

## Introduction

Sickle cell disease (SCD) is a hereditary hemoglobinopathy characterized by the presence of abnormal hemoglobin S (HbS), which leads to the sickling of red blood cells, chronic hemolytic anemia, and recurrent episodes of pain due to vaso-occlusion in the microcirculation [1]. This genetic disorder is especially prevalent in regions with high malaria endemicity, including parts of Africa, India, the Middle East, and the Mediterranean [2]. Recent studies have underscored the significant burden SCD places on affected populations, not only in terms of morbidity and mortality but also concerning its social and economic impacts [3]. In India, SCD poses a notable public health challenge, particularly among tribal populations and socially disadvantaged communities. Certain regions, such as Madhya Pradesh, Maharashtra, Odisha, and parts of Northeast India, report higher gene frequencies for the sickle cell trait (SCT) and SCD [4]. Estimates suggest that around 1.3 million individuals in India are affected by SCD, with the highest prevalence among tribal populations where SCT can reach up to 20% [5].

The clinical course of SCD varies widely among individuals and is influenced by both genetic and environmental factors [6]. In patients with SCD, vaso-occlusive crises (VOCs) are among the most common and debilitating complications, resulting in frequent hospitalizations and increased risk of organ damage [7]. A study by McGann et al. (2020) has highlighted the importance of hydroxyurea, a disease-modifying agent, in reducing the frequency and severity of VOCs and enhancing the quality of life for SCD patients [8]. Hydroxyurea, while increasingly adopted in India, remains underutilized due to cost, limited awareness, and lack of comprehensive healthcare access, especially in rural and tribal regions [9].

India’s "National Sickle Cell Anemia Elimination Mission" launched in 2023 aims to eliminate SCD as a public health problem by screening over 70 million people in high-prevalence areas, enhancing treatment access, and raising awareness [10]. However, the implementation of such large-scale health interventions faces several challenges, including limited healthcare infrastructure in rural areas, social stigma, and low disease literacy among affected communities [11]. In addition, while blood transfusions remain a cornerstone in managing severe anemia associated with SCD, frequent transfusions increase the risk of iron overload, necessitating chelation therapy. However, as Kanter and Kruse-Jarres (2020) observed, the financial burden of both transfusions and chelation agents like deferasirox often limits their consistent use in resource-limited settings [12].

Recent research has also emphasized the role of advanced therapeutic interventions such as hematopoietic stem cell transplantation (HSCT) and emerging gene therapies, which offer potential curative options for SCD [13]. Nonetheless, these therapies are currently limited by high costs, lack of donor availability, and potential adverse effects, thereby restricting their accessibility to a minority of patients globally. Non-curative, symptom-focused management remains the predominant approach, particularly in low- and middle-income countries [14]. Within this context, there is a critical need to better understand the demographic and clinical profiles of SCD patients in India to inform health policies and optimize care strategies tailored to local needs [15].

This study aims to provide a comprehensive analysis of SCD patients presenting with complications at a tertiary healthcare center in Upper Assam, India. By examining demographic characteristics, clinical features, treatment patterns, and healthcare challenges in this specific population, this study contributes to the growing body of literature on SCD in India and identifies areas for improving SCD management in resource-limited settings. It also aims to highlight disparities in access to advanced care options and the reliance on traditional management approaches such as hydroxyurea and blood transfusions. Understanding these patterns can support public health interventions and align with national efforts to address the complex burden of SCD in India.

## Materials and methods

Study site

This study was conducted at Assam Medical College and Hospital (AMCH), a tertiary healthcare center in Dibrugarh, Upper Assam, India. Data were obtained from medical records in the Departments of Medicine and Pediatrics, covering patients seen from January 1, 2020, to December 31, 2023.

Study population

The study included all patients diagnosed with sickle cell disease (SCD), including sickle cell anemia (Hg SS), sickle cell trait (Hg SA), sickle cell hemoglobin E trait (Hg SE), and sickle cell thalassemia. These patients attended AMCH specifically for complications related to SCD, such as vaso-occlusive crises, infections, and other systemic manifestations.

Inclusion criteria

The inclusion criteria for the study comprised of patients with confirmed SCD diagnosis (any of the genotypes specified above) and patients who presented with SCD-related complications requiring medical intervention who had complete medical records available for demographic, clinical, and treatment variables.

Exclusion criteria

Exclusion criteria for the study included patients with unrelated medical conditions who did not present for SCD complications and patients with incomplete or missing medical records, particularly concerning treatment details and clinical presentations.

Data collection

Data were extracted retrospectively from patient records, including demographic information (age, gender, and ethnicity), clinical features (specific complications, hemoglobin levels, genotype, history of hospitalizations), and treatment patterns (frequency of blood transfusions, hydroxyurea therapy, use of chelating agents). A structured proforma was used to ensure data consistency.

Methodology

The study involved a retrospective analysis of clinical records. Demographic, clinical, and treatment data were compiled, and each patient’s SCD-related complication history was assessed. The analysis focused on the prevalence of clinical symptoms, patterns in blood transfusion needs, and the utilization of therapeutic agents like hydroxyurea and chelating agents.

Ethics statement

This study received approval from the institutional review board of AMCH. All patient information was anonymized to maintain confidentiality and adhere to ethical standards.

Statistical analysis

Descriptive statistics summarized demographic and clinical characteristics. Continuous variables were expressed as means ± standard deviations, and categorical data were presented as frequencies and percentages. Bivariate analyses, including chi-square tests and t-tests, compared demographic and clinical variables between patient groups. Logistic regression was employed to identify factors associated with high transfusion needs. Statistical significance was set at p < 0.05.

## Results

**Figure 1:**
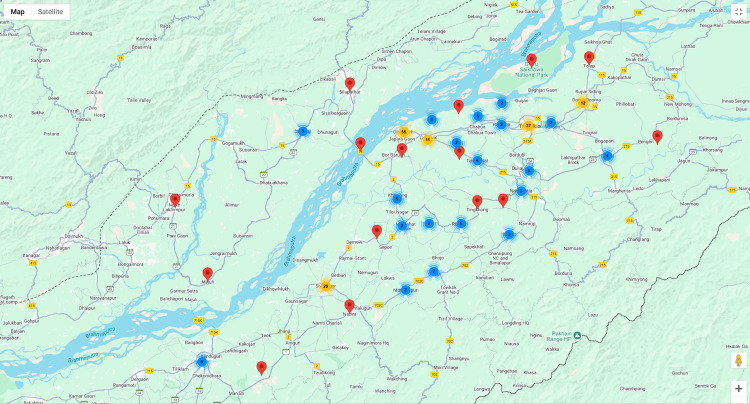
Geographic distribution of SCD pattients from Upper Assam region of North East India

A total of 250 patients were included in the study, with 216 (87%) diagnosed with sickle cell disease (Hg SS), 16 (6%) with sickle cell HbE, seven (3%) with sickle cell beta-thalassemia and 11 (4%) with sickle cell trait (Hg SA). In the present study, the geographical distribution of sickle cell anemia patients from the Upper Assam region was assessed. The majority of patients originated from Dibrugarh district, totaling 112 patients (44.8%), followed by 64 patients (25.6%) from Tinsukia district. Sivasagar and Charaideo districts contributed 32 (12.8%) and 19 (7.6%) patients, respectively. Jorhat district accounted for nine patients (3.6%), while Lakhimpur had three patients (1.2%) and Dhemaji had four patients (1.6%). Additionally, Golaghat, Nagaon, and Nalbari districts each contributed two patients (0.8%) (Figure 1). This distribution highlights the concentration of cases in districts closer to Dibrugarh, where Assam Medical College and Hospital, the study’s data collection center, is located, suggesting both a prevalence in the population and accessibility to healthcare services for diagnosis and treatment

The gender distribution was 136 (54.4%) males and 114 (45.6%) females. The mean age of the patients was 17.22 (+8.8) years. The majority of patients 125 (50%) were aged between 10 and 20 years, followed by 53 (21.2%) patients aged 20 to 30, 49 (19.6%) patients under the age of 10, and 23 (9.2%) patients over 30 (Table 1). Common clinical manifestations observed were fever in 153 patients (61.2%), pallor in 75 patients (30%), and bone and joint pain in 121 patients (48.4%). Other notable symptoms included abdominal pain 64 (25.6%), fatigue 69 (27.6%), and chest pain 30 (12%). Jaundice was reported in 29 (11.6%) patients, while 13 (5.2%) patients experienced dyspnoea. Less common symptoms included edema, which was noted in only six (2.4%) patients (Table 1).

**Table 1:** Demographic profile of SCD patients attending tertiary care centre

Sl no	Parameter	Number (n)	Percentage (%)
Gender Distribution			
1	Males	136	54.4
2	Females	114	45.6
Age distribution			
1	< 10 years	49	19.6
2	10 – 19 years	125	50
3	20 - 29 years	53	21.2
4	>30 years	23	9.2
Clinical Presentation			
1	Fever	153	61.2
2	Bone and joint pain	121	48.4
3	Pallor	75	30
4	Abdominal pain	64	25.6
5	Fatigue	69	27.6
6	Chest pain	30	12
7	Jaundice	29	11.6
8	Dyspnoea	13	5.2
9	Edema	6	2.4

The average hemoglobin (Hb) level was 7.19 (+2.37) g/dL, with a mean corpuscular volume (MCV) of 76.39 fL and a mean corpuscular hemoglobin (MCH) of 25.45 pg. Serum ferritin levels, indicating iron overload, had a mean value of 1.43 (+0.5) ng/mL, reflecting the varying degrees of iron overload. Bilirubin levels averaged 1.85 μmol/L, suggesting ongoing hemolysis, while creatinine levels (mean: 0.61 mg/dL) remained within normal limits for most patients, indicating preserved kidney function. The frequency of blood transfusions was a significant aspect of the study. The mean number of transfusions per year was 12.61, with a standard deviation of 7.06. Notably, 181 patients (72.4%) received between five to 12 transfusions annually, 40 (16%) patients received more than 12 blood transfusions per year, 10 (4%) patients received between three to five transfusions, and only three (1.2%) patients required one to two transfusions annually. Sixteen patients did not require any transfusion during the study period (Table 2).

**Table 2:** Laboratory reports and number of blood transfusions per annum in SCD patients MCV: mean corpuscular volume; MCH: mean corpuscular hemoglobin; MCHC: mean corpuscular hemoglobin concentration; RDW: red cell distribution width; RBC: red blood cell count; PLT: platelet count; HBS: hemoglobin S; HBA: hemoglobin A; HBF: hemoglobin F

LABORATORY PARAMETERS		Mean (+SD)
1	Haemoglobin (g/dl)	7.19 (+2.37)
2	MCV(fl)	76.39 (+10.74)
3	MCH (pg)	25.45 (+5.01)
4	MCHC (g/dl)	31.74 (+3.7)
5	RDW	22.52 (+6.57)
6	RBC Count (10^9^/L)	2.76 (+1.64)
7	PLT Count (10^9^/L)	202 (+108)
8	Reticulocyte Count (%)	2.68 (+1.8)
9	HBS (%)	65.92 (+11.35)
10	HBA (%)	5.45 (+4.13)
11	HBF (%)	15.71 (+8.17)
12	HBA2 (%)	3.77 (+2.7)
13	Serum Ferritin	1.43 (+0.5)
14	Serum Bilirubin (mg/dl)	1.85
15	Creatinine (mg/dl)	0.61 (+0.34)
Number of Blood transfusion per year		
1	0	16 (6.4%)
2	1-2	3 (1.2%)
3	3-5	10 (4%)
4	5-12	181 (72.4%)
5	>12	40 (16%)

The study examined the treatment regimen of hydroxyurea and chelating agents. Half of the patients (125) were on hydroxyurea therapy, while the other half did not receive it. Chelating agents, such as deferasirox or desferal, were administered to 56 patients (22.4%), primarily to manage iron overload from repeated blood transfusions. The remaining 194 patients (77.6%) were not on any chelation therapy (Table 2).

## Discussion

This study provides a detailed profile of sickle cell disease (SCD) patients presenting with complications in Upper Assam, a region with distinct demographic and healthcare challenges. The findings highlight the heavy burden of SCD in this setting, where limited access to healthcare and socioeconomic factors significantly impact the management and outcomes for affected individuals.

Demographic profile and disease burden

The study population had a mean age of 17.2 years, with the majority of patients in their teenage years. This is consistent with data from other Indian studies, which report similar age demographics in tribal populations [8]. These findings underscore that SCD symptoms often manifest early, prompting frequent hospital visits during adolescence due to severe complications like VOCs and infections. Globally, studies in Africa and the Middle East have observed a similar trend of pediatric and adolescent dominance in SCD hospitalizations, emphasizing the early onset of disease complications in regions with limited resources [14]. In high-income countries, however, better access to disease-modifying therapies like hydroxyurea and more comprehensive healthcare systems have allowed for improved life expectancy, shifting the disease burden toward older age groups [6].

Clinical features and complications

In our cohort, fever (61.2%) and bone/joint pain (48.4%) were the most common presenting symptoms, with other manifestations like pallor, abdominal pain, and jaundice observed to a lesser extent. Fever and VOCs are hallmark symptoms of SCD worldwide, driven by recurrent infections and the vaso-occlusive process associated with the sickling of red blood cells. A study in Nigeria similarly reported high rates of fever and pain as predominant symptoms, indicating the universal nature of these complications across diverse SCD populations [16]. While bone pain crises are well-recognized complications, the frequent association with fever suggests a high prevalence of infections, likely exacerbated by environmental factors and suboptimal prophylactic care in India [15]. In Western countries, the incidence of fever among SCD patients tends to be lower, which may reflect better prophylactic measures against infection [7].

Additionally, our study observed lower frequencies of respiratory complications such as chest pain and dyspnea, which are often indicators of acute chest syndrome (ACS), a serious SCD complication. In contrast, a study by Gladwin et al. [17] in the United States highlighted ACS as a frequent and severe manifestation in American SCD populations. The lower incidence of ACS in our study could be attributed to underreporting or differences in clinical recognition of respiratory symptoms in resource-limited settings. This finding emphasizes the need for enhanced diagnostic protocols to identify and manage such complications more effectively.

Treatment patterns: hydroxyurea, blood transfusions, and chelation therapy

Half of the patients in our study were on hydroxyurea, which aligns with the increasing acceptance of this drug in India as an effective therapy for reducing VOC frequency and transfusion needs. Hydroxyurea has been shown to significantly reduce the incidence of VOCs and hospitalizations by inducing fetal hemoglobin (HbF) production, which prevents HbS polymerization [18]. However, in our setting, hydroxyurea utilization is still limited by factors such as drug costs, inconsistent availability, and a lack of awareness. A study in the United States showed that over 80% of eligible SCD patients use hydroxyurea, indicating a stark contrast in access and uptake [10].

The study also reveals a high transfusion burden, with 72.4% of patients requiring 5 to 12 transfusions annually. Blood transfusions are critical for managing severe anemia and preventing stroke in SCD patients but come with risks such as alloimmunization and iron overload. In high-resource settings, transfusion frequency is generally lower due to more extensive use of hydroxyurea and other therapies [19]. Our findings align with a similar study from sub-Saharan Africa, where high transfusion rates highlight the lack of access to alternative therapies [20]. The iron overload from repeated transfusions is another concern, as indicated by the mean serum ferritin levels in our patients. Chelation therapy was administered to only 22.4% of patients, far lower than international recommendations, due to cost and availability issues [1].

Comparisons with Indian studies

Compared to other Indian studies, our findings are consistent with reports of high transfusion dependence and underuse of hydroxyurea and chelating agents in tribal and rural populations. A study from Gujarat echoed the challenge of iron overload due to transfusion reliance, underscoring the need for accessible chelation options in India’s rural settings [21]. Additionally, our study is one of the few from Northeast India, offering unique insights into SCD management in a historically underserved region. This regional focus is significant, as Assam’s tea tribe communities, descendants of labor migrants, exhibit a high prevalence of SCD and face substantial healthcare disparities [22].

Limitations and future directions

This study’s retrospective nature and reliance on medical records may have introduced information bias, especially given the possibility of incomplete documentation in patient files. As a single-center study, the findings may not be generalizable to all Indian SCD populations, particularly those in urban settings with better healthcare access. Future multicenter studies with prospective follow-up can provide a more comprehensive understanding of SCD complications and treatment outcomes in diverse settings across India. Recent advancements, including gene therapy and bone marrow transplantation, represent promising curative approaches for SCD [23]. However, their high cost and limited availability restrict their use in India and other low-resource countries, highlighting an urgent need for feasible, accessible treatment options to reduce the disease burden in these regions.

## Conclusions

In summary, our study contributes valuable data on the demographic and clinical characteristics of SCD patients in Upper Assam, with findings that mirror the challenges of SCD management in resource-limited settings. The study underscores a critical need for enhanced access to hydroxyurea, transfusion alternatives, and chelation therapy in the region. As India implements its National Sickle Cell Anemia Elimination Mission, such localized studies are essential to inform policies and tailor interventions to the needs of specific populations, ultimately improving the quality of life for individuals with SCD.
